# Association of Platelet Desialylation and Circulating Follicular Helper T Cells in Patients With Thrombocytopenia

**DOI:** 10.3389/fimmu.2022.810620

**Published:** 2022-04-01

**Authors:** Yuwen Chen, Liping Luo, Yongzhi Zheng, Qiaoyun Zheng, Na Zhang, Donghui Gan, Shimuye Kalayu Yirga, Zhenxing Lin, Qizhen Shi, Lin Fu, Jianda Hu, Yingyu Chen

**Affiliations:** ^1^ Department of Hematology, Fujian Institute of Hematology, Fujian Provincial Key Laboratory of Hematology, Fujian Medical University Union Hospital, Fuzhou, China; ^2^ Department of Pediatrics, Medical College of Wisconsin, Milwaukee, WI, United States; ^3^ Blood Research Institute, Versiti, Milwaukee, WI, United States; ^4^ Department of Hematology, The Second Affiliated Hospital of Guangzhou Medical University, Guangzhou, China

**Keywords:** thrombocytopenia, platelet, desialylation, follicular helper T Cells (TFHs), CXCL13

## Abstract

Thrombocytopenia is a multifactorial condition that frequently involves concomitant defects in platelet production and clearance. The physiopathology of low platelet count in thrombocytopenia remains unclear. Sialylation on platelet membrane glycoprotein and follicular helper T cells (TFHs) are thought to be the novel platelet clearance pathways. The aim of this study was to clarify the roles of platelet desialylation and circulating TFHs in patients with immune thrombocytopenia (ITP) and non-ITP thrombocytopenia. We enrolled 190 patients with ITP and 94 patients with non-ITP related thrombocytopenia including case of aplastic anemia (AA) and myelodysplastic syndromes (MDS). One hundred and ten healthy volunteers were included as controls. We found significantly increased desialylated platelets in patients with ITP or thrombocytopenia in the context of AA and MDS. Platelet desialylation was negatively correlated with platelet count. Meanwhile, the circulating TFH levels in patients with thrombocytopenia were significantly higher than those of normal controls, and were positively correlated with desialylated platelet levels. Moreover, TFHs-related chemokine CXCL13 and apoptotic platelet levels were abnormally high in ITP patients. The upregulation of pro-apoptotic proteins and the activation of the MAPK/mTOR pathway were observed in the same cohort. These findings suggested that platelet desialylation and circulating TFHs may become the potential biomarkers for evaluating the disease process associated with thrombocytopenia in patients with ITP and non-ITP.

## Introduction

Thrombocytopenia is a condition characterized by abnormally low platelet levels. Several diseases present with thrombocytopenia, such as immune thrombocytopenia (ITP), aplastic anemia (AA), leukemia, or myelodysplastic syndrome (MDS). Severe thrombocytopenia predisposes patients to spontaneous bleeding. Among these, ITP is associated with the production of anti-platelet autoantibodies, which may increase the destruction of platelets and impair platelet production. Previous studies have found that platelet glycoproteins (GP)IIb-IIIα and GPIb-IX are the two most frequently targeted autoantigens in ITP patients (1–3). Autoantibodies against platelet GPIIb-IIIa may clear the platelets through Fc-FcγR interactions *via* the reticuloendothelial system (RES). GPIb-IX, on the other hand, may be associated with Fc-independent platelet activation, and is closely related to the progress of platelet desialylation (2, 4, 5). The surfaces of platelets are covered with abundant sialic acid, which is gradually removed by sialidases (like Neuraminidase 1 and Neuraminidase 3) as platelets circulate in the peripheral blood. This progress is known as platelet desialylation (4, 6). Studies further showed that anti-GPIbα-mediated-desialylation and platelet activation can lead to FcγR-independent clearance *via* Ashwell Morell receptor (AMR) in hepatocytes (4, 7, 8). To date, knowledge of the underlying mechanisms of platelet desialylation in thrombocytopenia remains limited, and it is still unclear whether platelet desialylation may serve as a diagnostic or prognostic biomarker of the disease. Thus, the extensive investigation will likely unravel significant benefits for patients with thrombocytopenia.

Follicular helper T cells (TFHs) are a novel distinct CD4+ T cell subset which takes part in the activation and differentiation of B cells. TFHs can migrate to target organs, and secrete cytokines to mediate the occurrence of autoimmune diseases (5, 7). Audia, Rossato (5) showed that B cell depletion after rituximab (RTX) treatment in ITP patients led to a dramatic decrease of circulating TFHs. TFHs express specific surface markers, such as CXCR5, programmed cell death protein 1 (PD-1) and inducible co-stimulator (ICOS) (9). CXCR5 is the landmark surface marker of TFHs. It can bind to CXCL13 and then assist B cells in the migration to the lymphatic follicular area for homing volatilization (10, 11). CXCL13 is the only ligand of CXCR5. The interaction between CXCL13 and CXCR5 plays a critical part in the process of lymphocyte migration and formation of the germinal center (GC). It has been reported that CXCL13-CXCR5 axis is closely associated with the occurrence of dozens of diseases, including rheumatoid arthritis (12, 13), systemic lupus erythematosus (14, 15), prostatic cancer (16), colorectal cancer (17) and mammary cancer (18). However, limited data are available on the roles of CXCL13 and the circulating CXCR5+ TFHs in patients with thrombocytopenia. In-depth investigation into these is of great value to exploit novel biomarker as potential therapeutic target for thrombocytopenia.

Apoptosis is another main pathway of platelet clearance, playing a critical role in the life-span and function of circulating platelets. This process is associated with homeostasis and various pathophysiological progresses (19–21). Thus, the relationship between platelet apoptosis and thrombocytopenia merits further study. Here, we detected the proportion of desialylated platelets in the peripheral blood of patients with ITP and non-ITP related thrombocytopenia. And then we measured circulating TFH levels and analyzed the correlation between TFHs and desialylated platelets in patients with thrombocytopenia. Furthermore, we explored plasma CXCL13 levels and platelet apoptosis and evaluated the associations of platelet desialylation and apoptosis in patients with ITP. Finally, we investigated the expression changes of pro-apoptotic proteins and MAPK/mTOR pathway in the same cohort. In addition, we further addressed the correlation of platelet desialylation with treatment response. The present findings may provide the valuable biomarkers for evaluating the disease process associated with ITP and non-ITP related thrombocytopenia.

## Materials and Methods

### Patient Demographics

Using the criteria of the International Working Group of ITP and the Chinese guidelines on the diagnosis and management of primary ITP in adults (22, 23), we analyzed 190 patients diagnosed with ITP in Fujian Medical University Union Hospital. Patients with non-ITP related thrombocytopenia including cases of AA (n = 68) and MDS (n = 26) were also enrolled in this study. The clinical records of patients were independently reviewed by two physicians. In assessing the efficacy of treatment, complete response (CR) was defined as a platelet count ≥ 100 × 10^9^/L. Response was defined as a platelet count ≥ 30 × 10^9^/L or a doubling of baseline platelet count within 7 days. A total of 110 healthy volunteers were included as controls. Ethics approval was obtained from the Institutional Review Board of Fujian Medical University Union Hospital (2021KY152). The demographic and clinical characteristics of the patients and healthy controls are presented in **Table 1**.

**Table 1:** Demographic and clinical characteristics in entire thrombocytopenia cohort and healthy controls.

Characteristics	Thrombocytopenia	Healthy controls		P-value
**ITP**				
n	190	110	–	
Age, y	35 [30, 55]	36 [27, 44]	0.08	
Female/Male	129/61	75/35	0.96	
PLT(×10^9^/L)	51 [18, 101]	230 [201, 261]	<0.00	
Disease duration, m	2 [0, 10]	–	–	
**AA**				
n	68	77	–	
Age, y	36 [25, 52]	36 [29, 45]	0.67	
Female/Male	28/40	55/22	0.00	
PLT(×10^9^/L)	33 [13, 85]	229 [197, 259]	<0.00	
Disease duration, m	15 [4, 31]	–	–	
**MDS**				
n	26	33	–	
Age	49 [42, 66]	34 [26, 51]	0.00	
Female/Male	14/12	23/10	0.21	
PLT(×10^9^/L)	41 [17, 126]	235 [197, 257]	<0.00	
Disease duration, m	1.5 [0.6, 9.5]	–	–	

PLT, platelet; ITP, immune thrombocytopenia; AA, aplastic anemia; MDS, myelodysplastic syndromes; y, year; m, month.

### Cell Isolation and Platelet Preparation

Peripheral whole blood samples were obtained at patients’ scheduled visits after disease diagnosis. Peripheral blood mononuclear cells (PBMCs) were isolated according to the procedures we previously reported (24–26). For platelet preparation, venous blood was obtained by venipuncture into EDTA-based anticoagulant tubes. Platelet-rich plasma (PRP) was prepared as previously described (27).

### Flow Cytometry Analysis

Fluorescein-conjugated Ricinus Communis agglutinin I (RCA-I, Vector Laboratories, Burlingame, CA, USA) and Erythrina cristagalli lectin (ECL, Vector Laboratories, Burlingame, CA, USA) were used to detect desialylated galactose and β-GlcNAc residues *via* flow cytometry (BD FACSVerse™, Becton Dickinson, CA, USA) according to previously reported methods (28). For apoptosis analysis, platelet staining was performed using Annexin V Apoptosis Detection Kit (BectonDickinson, CA, United States) and analyzed by flow cytometry. For the measurement of circulating TFHs, PBMCs were incubated with the following fluorophore-conjugated antibodies: anti-CD4 (PerCP-Cy™ 5.5), anti-CD185 (CXCR5, PE), and anti-CD279 (PD-1, APC). These antibodies were obtained from BioLegend. All the staining procedures were performed according to the manufacturer’s protocol. All samples were analyzed with a flow cytometer. The data obtained were processed with Flowjo software, version 10 (TreeStar, Inc.).

### Chemokine Measurement

For chemokine measurement, plasma supernatant was isolated from the peripheral whole blood obtained from ITP patients and the healthy donors. The plasma samples were stored in Eppendorf centrifuge tubes at -80°C until use. The level of CXCL13 expression was assessed by an enzyme-linked immunosorbent assay (ELISA) following the protocol provided by the manufacturer (SimpleStep ELISA Kit, Abcam).

### Detection of Platelet Antibodies

The circulating platelet autoantibodies in the plasma of ITP patients were tested according to the protocol of Lifecodes Pakauto assay (GTI-PAKAUTO kit; Beijing BO FU RUI Gene Diagnostics). These samples were added to microwells coated with platelet glycoproteins allowing antibody binding. Unbound antibodies were then washed away. An alkaline phosphatase-labeled anti-human globulin reagent (anti-IgG/A/M) was added and incubated. The unbound antibodies were washed away and the substrate p-nitrophenyl phosphate (PNPP) was added. After a 30-minute incubation period, the reaction was stopped with Stopping Solution. The absorbance at 409 nm and 490 nm was measured using a Molecular Devices SpectraMax iD5 plate reader.

### Western Blotting

Proteins were isolated from the platelets, using Western blotting analysis, which was performed according to procedures previously reported (29). Primary antibodies against phospho-mTOR, mTOR, phospho-MAPK, MAPK, Bax, Bak and Bcl-2 were purchased from Cell Signaling Technology (Danvers, MA, United States). GAPDH antibody was obtained from Abcam (Cambridge, United Kingdom). Fluorescent signals were detected using the ChemiDoc Touch Imaging System (Bio-Rad, USA).

### Statistical Analysis

Statistical significance between experimental groups was analyzed by Mann-Whitney test or one-way ANOVA. Data are given by median [interquartile range]. Differences between qualitative variables were assessed with Pearson’s Chi-square test. Spearman correlation coefficients were calculated to evaluate the association between two continuous variables. Receiver Operating Characteristic curve (ROC) was used to evaluate the accuracy of ECL and RCA-I value in distinguishing the thrombocytopenia patients. A *P-*value of less than 0.05 was considered statistically significant.

## Results

### Platelet Desialylation in ITP and Non-ITP Thrombocytopenia Patients

**Figure 1:**
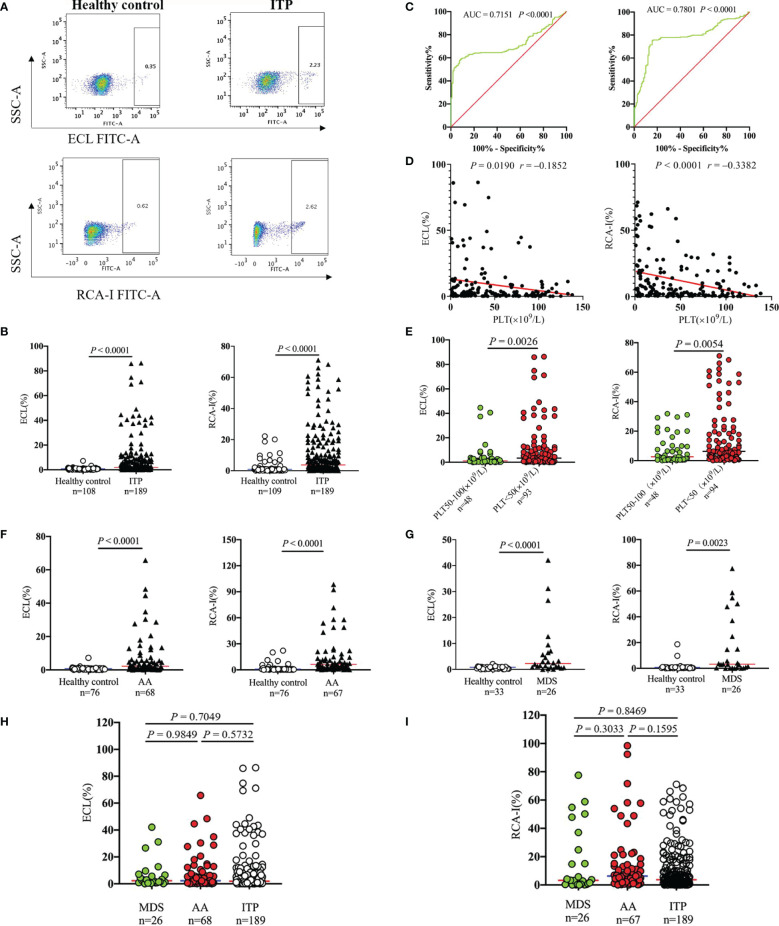
Platelet desialylation in patients with immune thrombocytopenia (ITP) and non-ITP related thrombocytopenia. **(A)** ECL or RCA-I binding to platelets were examined by flow cytometry; representative dot plots from healthy control and ITP patients are shown. **(B)** Comparison of platelet desialylation levels between ITP patients and healthy controls. **(C)** ROC curve for predicting platelet desialylation in ITP patients based on ECL (left) and RCA-I (right) binding methods. **(D)** Correlation analysis between platelet desialylation and platelet count (<150×10^9^/L) as determined by Spearman’s test. **(E)** Comparison of desialylated platelet levels in ITP patients with different platelet count. **(F, G)** Platelet desialylation levels in patients with aplastic anemia (AA), or myelodysplastic syndrome (MDS) versus healthy controls. **(H, I)** Comparison of desialylated platelet levels among AA, MDS, and ITP groups. ECL, Erythrina cristagalli lectin; RCA-I, Ricinus communis agglutinin I; ROC, Receiver operating characteristic.

Platelet desialylation levels were tested in the randomly enrolled patients diagnosed with ITP and healthy volunteers. Results showed that desialylated platelet levels of ITP patients were 2.2- and 4.2-fold higher than those of healthy volunteers as measured by ECL and RCA-I binding, respectively (1.93% [0.53-7.44] *vs*. 0.88% [0.35-0.98], *P* < 0.0001; 3.71% [1.24-15.00] *vs*. 0.88% [0.28-1.00], *P* < 0.0001; **Figures 1A, B** **)**. The area under the receiver operating curve (ROC-AUC) analysis suggested the prognostic value of ECL (AUC = 0.7151) and RCA-I (AUC = 0.7801) in ITP (**Figure 1C**). Moreover, Spearman correlation analysis showed that platelet desialylation was negatively correlated with platelet count (r = -0.1852, P = 0.0190 for platelet count vs ECL ratio; and r = -0.3382, P < 0.0001 for platelet count vs. RCA-I ratio, respectively; **Figure 1D**). ITP patients with higher levels of desialylated platelets exhibited significantly lower platelet count (**Figure 1E**).

The platelet desialylation was further evaluated in patients with non-ITP related thrombocytopenia, AA and MDS. Interestingly, we also observed dramatically increased platelet desialylation as measured by both ECL and RCA-I binding method (AA: 2.24% [0.61-6.55] *vs*. 0.84% [0.36-0.96], *P* < 0.0001 for ECL binding; 6.26% [2.06-14.40] *vs*. 0.87% [0.27-1.00], *P* < 0.0001 for RCA-I binding. MDS: 2.28% [0.93-6.26] *vs*. 0.81% [0.25-0.95], *P* < 0.0001 for ECL binding; 3.24% [0.41-22.35] *vs*. 0.74% [0.19-0.98], *P* = 0.0023 for RCA-I binding; **Figures 1F, G** **)**. However, there were no significant differences when compared the desialylated platelet levels among ITP, AA and MDS groups (**Figures 1H, I**). These data indicate that the existence of platelet desialylation can be used as a potential diagnostic and prognostic biomarker for thrombocytopenia.

### The Role of TFHs in ITP and Non-ITP Thrombocytopenia Patients

**Figure 2:**
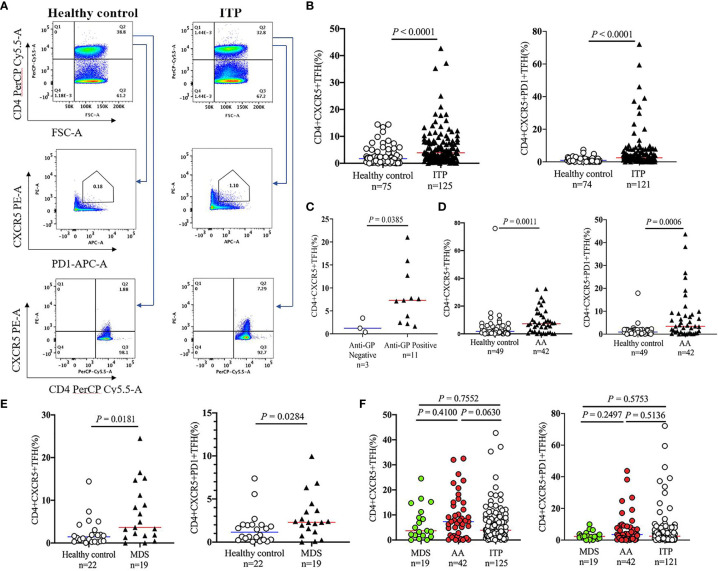
Circulating TFH levels in peripheral blood of patients with thrombocytopenia. Peripheral blood mononuclear cells (PBMCs) were isolated from patients with ITP, and were stained with labeled antibodies. PBMCs from healthy volunteers were used as controls in parallel. **(A)** Representative flow cytometry plots of CD4+CXCR5+PD1+ TFHs or CD4+CXCR5+ TFHs are depicted. **(B)** Frequencies of CD4+CXCR5+PD1+ TFHs and CD4+CXCR5+ TFHs in ITP patients versus controls. **(C)** Comparison of CD4+CXCR5+ TFH levels in ITP patients with the platelet glycoprotein antibodies versus those without antibodies. **(D, E)** TFH levels in patients with AA, MDS versus healthy controls. **(F)** Frequencies of circulating TFHs among patients with AA, MDS, and ITP. TFHs, T follicular helper cells.

TFHs represent a new subset of CD4+ T cells, which were involved in the development of pathologies and immunopathogenesis of several diseases (30–32). As the detailed roles of TFHs in patients with thrombocytopenia remain unclear, the circulating TFHs were then analyzed by flow cytometry in this study. We found a 2.3- and 2.7-fold increase of CD4+CXCR5+ TFHs and CD4+CXCR5+ PD1+ TFHs in ITP patients compared to healthy controls (3.88% [1.63-7.89] *vs*. 1.71% [0.49-2.78], *P* < 0.0001, **Figure 2A**; 2.47% [1.01-5.66] *vs*. 0.90% [0.31-1.97], *P* < 0.0001, **Figure 2B**). In addition, patients bearing anti-platelet autoantibodies displayed 5.9-fold higher levels of TFHs than those without autoantibodies (*P* = 0.0385; **Figure 2C**). The frequencies of TFHs were further investigated in non-ITP related thrombocytopenia. As compared with normal controls, CD4+CXCR5+ TFHs and CD4+CXCR5+PD1+ TFHs were markedly elevated in patients with AA (7.33% [1.69-13.23] *vs*. 1.82% [0.60-5.22], *P* = 0.0011 and 3.44% [0.78-8.49] *vs*. 0.96% [0.56-1.92], *P* = 0.0006, respectively; **Figure 2D**) or MDS (3.65% [1.80-10.05] *vs*. 1.44% [0.44-2.75], *P* = 0.0181 and 2.29% [1.38-3.58] *vs*. 1.15% [0.33-1.99], *P* = 0.0284, respectively; **Figure 2E**). But there were no significant differences when we further compared the TFH levels among ITP, AA and MDS groups (**Figure 2F**). These results indicate that the expansion of TFHs is particularly involved in the immunopathogenesis of ITP or non-ITP thrombocytopenia.

### Correlation Analysis Between Platelet Desialylation and TFHs in ITP

**Figure 3:**
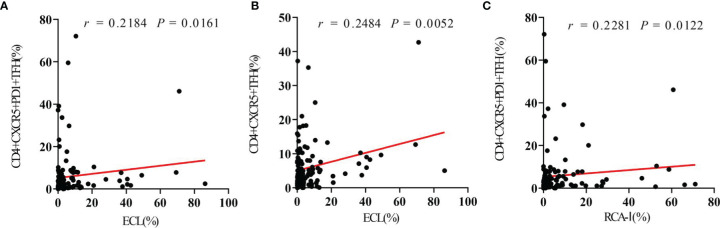
Circulating TFHs positively correlate with platelet desialylation in ITP patients. **(A)** Correlation between circulating CD4+CXCR5+PD1+ TFHs and ECL binding. **(B)** Correlation between circulating CD4+CXCR5+ TFHs and RCA-I binding. **(C)** Correlation between circulating CD4+CXCR5+PD1+ TFHs and RCA-I binding. Spearman’s rank correlation coefficient (r) and *P*-value are depicted.

Spearman correlation analysis was used to evaluate the relationship between platelet desialylation and TFHs in ITP patients. We observed a strong positive correlation between desialylated platelet level and TFH frequency (r = 0.2184, *P* = 0.0161 for ECL *vs*. CD4+CXCR5+PD1+ TFHs; r = 0.2484, *P* = 0.0052 for ECL *vs*. CD4+CXCR5+ TFHs; r = 0.2281, *P* = 0.0122 for RCA-I *vs*. CD4+CXCR5+PD1+ TFHs; **Figures 3A–C**). Higher levels of desialylated platelets were correlated with more frequencies of TFHs in the peripheral blood of ITP patients.

### ITP Patients Highly Express With the Chemokine CXCL13

**Figure 4:**
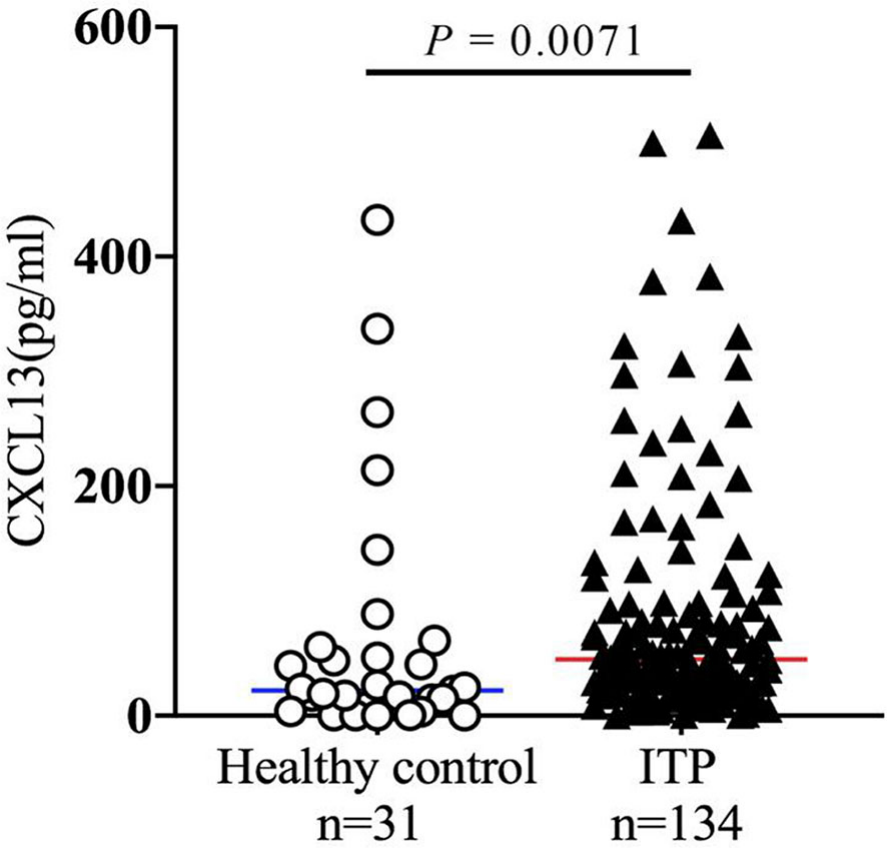
Chemokine CXCL13 expression in patients with ITP. Plasma samples were isolated from healthy volunteers and ITP patients. The plasma concentration of CXCL13 was quantified by ELISA. ELISA, enzyme-linked immunosorbent assay.

CXCL13, a cytokine mainly produced by TFHs, may promote the B cell maturation into antibody producing-plasma cells (33, 34). As high proportion of circulating CXCR5+ TFHs were found in ITP patients, and CXCR5 is the only receptor of CXCL13, we hypothesized that the cytokine CXCL13 might be upregulated in ITP patients as well. To further explore the role of this cytokine in the immunopathogenesis of ITP, the expression levels of plasma CXCL13 from a total of 134 ITP patients were measured by ELISA. As we expected, the CXCL13 levels in the ITP cohort were significantly increased by 2.3-fold compared to those in healthy controls (49.24 pg/ml [25.19-96.44] *vs*. 21.82 pg/ml [7.91-55.29], *P* = 0.0071; **Figure 4**), suggesting that chemokine CXCL13 was closely related with the occurrence of ITP. The characteristics of ITP patients and healthy controls are shown in **Table 2**. There were no significant differences in the age and sex distributions between ITP and healthy control groups.

**Table 2:** Demographic and characteristics for CXCL13 measurement in ITP cohort and healthy controls.

Characteristics	ITP (n=134)	Healthy controls (n=31)	P-value
Age, y	39 [30,56]	37 [31, 52]	0.61
Female/Male	90/44	22/9	0.68
PLT(×10^9^/L)	46 [16, 95]	230 [196, 276]	<0.00

PLT, platelet; ITP, immune thrombocytopenia.

### Platelet Apoptosis in ITP Patients

**Figure 5:**
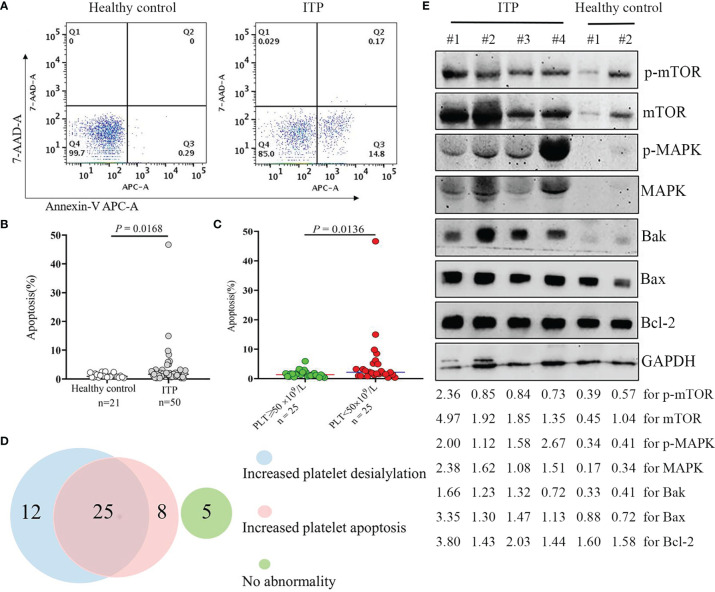
Platelet apoptosis in the ITP cohort. Apoptotic platelets were stained with Annexin V-APC/7-AAD, and analyzed by flow cytometry. **(A)** The representative dot plots from healthy controls and ITP groups are shown. **(B)** Comparison of platelets undergoing apoptosis in healthy controls and patients with ITP. The *P-*value was calculated using the Mann-Whitney test. **(C)** Comparison of apoptotic platelet levels in ITP patients with different platelet count. **(D)** Venn diagram showing the numbers of patients presenting with increased platelet apoptosis and/or increased platelet desialylation. **(E)** Protein extracts from isolated platelet lysate were subjected to Western blotting using phosphorylated mTOR and MAPK, total mTOR and MAPK, pro-apoptotic Bak and Bax, anti-apoptotic Bcl-2, and GAPDH antibodies. Four representative samples from ITP patients and two representative samples from healthy volunteers are shown. The intensity of different protein bands was quantified and normalized with GAPDH. Relative intensity of p-mTOR, mTOR, p-MAPK, MAPK, Bak, Bax and Bcl-2 is depicted by the lower set of numbers.

We further investigated the role of apoptosis in the reduction of platelet number in patients with thrombocytopenia. A total of 50 patient samples were randomly selected from ITP cohort, and 21 healthy control samples were included for platelet apoptosis measurement. As shown in **Figures 5A, B**, the apoptotic platelet levels were markedly increased by 1.8-fold in the ITP group in comparison with the healthy control group (*P* = 0.0168). Moreover, we found that apoptotic platelet levels were significantly associated with platelet count in ITP patients; the higher the levels of apoptotic platelets, the lower the platelet count in ITP cohort (**Figures 5C**). This may also be one possible explanation for the low platelet number in ITP patients, and these data are consistent with the results of previous study (35). The characteristics and the variables investigated in ITP cohort and healthy controls are shown in **Supplementary Table S1**.

### The Coexistence of Platelet Apoptosis and Desialylation in Patients With ITP

To better understand the contribution factors leading to thrombocytopenia in patients with ITP, we analyzed the proportion of patients with increased platelet apoptosis and desialylation (as evaluated by ECL or RCA-I binding). As shown in **Figures 5D**, half of the 50 ITP patients (50%) had both increased platelet apoptosis and desialylation, while 40% of them showed an increase in either apoptosis (8/50) or desialylation (12/50). Interestingly, five out of the 50 patients (10%) displayed none of the two abnormalities. Our findings further evidenced that both apoptosis and desialylation could be responsible for the reduction of platelet number in patients with thrombocytopenia.

### The Activation of MAPK/mTOR Pathway in ITP

We then looked at the underlying mechanisms linked to the platelet apoptosis induction in thrombocytopenia. Results showed that the expression levels of MAPK and mTOR and their phosphorylated proteins were dramatically up-regulated in the platelets of ITP patients as compared with healthy controls. Accordingly, the expressions of proapoptotic proteins Bak and Bax were increased, and the ratios of Bak/Bcl-2 and Bax/Bcl-2 were upregulated in the ITP patient samples, as shown by Western blotting analysis of platelet lysate (**Figures 5E**, **Supplementary Table S2**). These data suggested that the activation of the MAPK/mTOR pathway in response to the induction of platelet apoptosis is involved in platelet clearance in ITP.

## Discussion

The physiopathology of low platelet count in ITP and non-ITP related thrombocytopenia remains unclear. Glycoproteins on the surface of the platelet membrane are connected with sialic acid, which can be specifically bound to sialidase. The N-acetylglucosamine (GlcNac) and galactose (Gal) residues are exposed by this process, which can be recognized by integrin αMβ2 or AMR. In particular, platelets can be cleared in the liver afterwards (4, 7, 8). Li, van der Wal (4) showed that anti-GPIbα-mediated desialylation led to FcγR-independent platelet clearance *via* hepatocytes, and sialidase inhibition could rescue thrombocytopenia in the murine model of ITP. While sialylation on O-glycans may protect platelets from clearance by liver Kupffer cells (36). To date, very limited data are available regarding the role of desialylated platelets in patients with thrombocytopenia. As such, in the present study, we had assessed to blood samples from a cohort of 284 patients with thrombocytopenia and 110 healthy volunteers. We found more than 2.0-fold increase of the desialylated platelet levels in patients with ITP and non-ITP thrombocytopenia; and desialylated platelet frequencies were negatively correlated with platelet count. Therefore, from a clinical perspective, our data provide important insights, showing desialylation contributing to low platelet count in thrombocytopenia.

The association of desialylated platelets with circulating TFHs in thrombocytopenia was further addressed in this study. Audia, Rossato (5) reported splenic TFH expansion was involved in the production of antiplatelet-antibodies in patients with ITP. As splenic TFHs from human cannot obviously be assessed, and functional subpopulations of TFHs can be identified based on their specific markers such as PD-1, ICOS and CCR7 (37). In addition, PD-1 regulates GC positioning and definitely controls the functions of TFHs (38). We then defined the circulating TFHs as CD4+CXCR5+ TFHs and CD4+CXCR5+PD1+ TFHs in this study. Of note, we observed abnormal increase of both TFHs in the peripheral blood in ITP cohort. Patients bearing anti-platelet autoantibodies exhibited higher levels of TFHs than those without autoantibodies, suggesting TFHs might be potential indicator for immunosuppressive therapy in ITP. Given that the persistence of memory TFHs in secondary lymphoid organs (5, 39, 40), our data provide the speculative hint that monitoring of peripheral blood TFHs is feasible for evaluating disease progression. Furthermore, to the best of our knowledge, we are the first to demonstrate circulating TFHs expansion in non-ITP thrombocytopenia patients with MDS or AA. Importantly, we show the strong positive correlation between elevation of circulating TFHs and desialylated platelets, supporting TFHs as a critical mediator in synergy to promote platelet desialylation. A recent study found that H. Pylori infection may lead to the upregulation of TFHs in gastritis patients (41), and several recent reports revealed an association between H. Pylori infection and the incidence of ITP (42–44). Hence, we speculate that H. Pylori may be related to the abnormally elevated TFHs in the infected individuals with ITP in the present study. Future mechanistic studies are therefore warranted to systematically substantiate these findings.

Recent studies revealed that CXCL13-CXCR5 axis may change the signal transduction pathways and regulates the maturation and differentiation of B cells afterward; the transcriptional regulator of CXCL13 expression in T cells could become the primary target to improve anti-PD-1 immunotherapy (45–47). Intriguingly, we observed significant elevation of circulating CXCR5+ TFHs and plasma CXCL13 in thrombocytopenia cohort. As we know, CXCL13 is predominantly produced by GC TFHs (10, 11, 48), our results further reinforce the potential role of TFHs to be a new target for the treatment of thrombocytopenia. In addition, since CXCL13 may function as an available biomarker of GC activity in human vaccine trial and advanced stage disease (e.g. neuromyelitis optica spectrum disorders and chronic lymphocytic leukemia) (30, 49, 50), our findings suggest that plasma CXCL13 may act as another prognostic marker in patients with thrombocytopenia. Of note, analysis of plasma CXCL13 is not a disease- or antigen-specific readout. It is advisable using CXCL13 together with other biomarkers associated with the immunopathological setting in subsequent study.

Platelet lifespan in circulation is around 10 days in human (51). The stability and activity of circulating platelets are strictly regulated by the process of apoptosis. Zhao, Liu (52) showed that the activation of protein kinase A protected platelets from apoptosis and elevated the levels of peripheral platelets in murine ITP model. Chen, Yan (35) reported Akt activation and Akt-mediated platelet apoptosis in ITP patients with anti-GPIbα autoantibodies. Inhibition of Akt or Akt-regulated apoptotic signaling rescued platelets from clearance. In line with previous studies (35, 53), we found significantly increased apoptotic platelets appeared in thrombocytopenia cohort. More platelets undergoing apoptosis, and inferior platelet count was observed. Furthermore, we observed the remarkable activation of MAPK and mTOR occurred in platelet lysate from patients with thrombocytopenia. Accordingly, compared to healthy controls, platelets from thrombocytopenia patients exhibited enhanced expressions of pro-apoptotic proteins Bak and Bax. The ratios of Bak/Bcl-2 and Bax/Bcl-2 were consistently increased in this cohort. Thus, we provided a mechanistic insight into the pathway involved in apoptosis-mediated platelet clearance in thrombocytopenia.

Nevertheless, we gained further insight into the possible association between platelet apoptosis and desialylation in this study. It has been reported by Grodzielski, Goette (54) that platelet apoptosis and desialylation did not differ between patients undergoing treatment for ITP and those that were not being treated. They showed that megakaryopoiesis, platelet apoptosis and platelet desialylation did not follow a specific pattern in ITP patients. A recent study showed that changes in glycoside composition of glycoproteins on the platelets’ surface impaired the functional capacity of platelets and increased their apoptosis, which may be related to ITP severity (55). Our data showed that half of the samples (50%) exhibited both abnormalities; 24% and 16% of these samples presented only increased apoptosis or desialylation, respectively; while 10% of them behaved normally. These results reinforce the notion that there is no particular common factor leading to thrombocytopenia.

Platelet desialylation is also interestingly found in patients with non-ITP thrombocytopenia including AA and MDS. Currently, whether desialylation influence the platelet production in bone marrow (BM) remains further investigation. As shown in **Supplementary Figure S1**, patients (ITP, AA and MDS) with high levels of desialylated platelets (> 20% of ECL or RCA-I ratio) exhibit impaired megakaryocyte differentiation and maturation. However, the similar platelet production defects in megakaryocytes also exhibit in the BM of thrombocytopenia patients with or without low desialylation. A recent study reported that desialylating autoantibody (AAb) may contribute to the impaired platelet lifespan in an apoptosis-independent way, and interferes with the interaction between cells and extracellular matrix proteins leading to impaired platelet adhesion and megakaryocyte maturation (56). This finding makes it very likely that our observation among patients with thrombocytopenia was modulated by the sialylation status of the AAb.

The correlation between platelet desialylation and the therapeutic response in patients with ITP were further addressed in this study. As shown in **Supplementary Figure S2**, we found markedly decreased desialylated platelets in the included 29 ITP patients who achieved a platelet count CR to the first-line therapy. Correlation analysis revealed that the desialylated platelet level was negatively correlated with the platelet count in the peripheral blood. To date, there are 34 patients with ITP participated in the study who have completed 12 months’ follow-up. A total of 18 patients achieved CR to first-line therapy. As compared to CR group, 16 non-responders had significantly higher levels of platelet desialylation (ECL: *P* = 0.018; RCA: *P* < 0.0001). The higher desialylation, the poorer efficacy of therapy observed. Even though some patients switched to second-line drug treatment, we did not find the platelet count response among those patients at consecutive scheduled visits within 12 months. Based on the current clinical data, we confirm that platelet desialylation act as an important biomarker in determining response to treatment for patients with ITP. In agreement with previous reports (4, 57), our findings indicate the importance of developing strategies, such as sialylation inhibitors, in the treatment of ITP.

Whether sex may affect the presence of desialylation and TFHs has been evaluated in the study. We compared the desialylated platelet and TFHs levels between female and male in ITP cohort or healthy controls. Results demonstrated that either desialylation or TFH level was independent of sex in ITP patients and healthy controls **(** **Supplementary Table S3** **)**. A recent study also showed that RCA ratio in a population of 127 healthy subjects was not difference according to the sex (58), which is consistent with our finding. There are some limitations to the current study. First, more comprehensive mechanistic insights regarding the association of sialylation and thrombocytopenia require further elucidation. Second, the impressive numbers of patients have been included in the study, and long-term follow-up is still ongoing.

In summary, we demonstrated the abnormal increase of platelet desialylation and circulating TFHs in patients with thrombocytopenia. Desialylated platelet levels were positively associated with the expansion of TFHs, and were negatively correlated with platelet count. These findings revealed the potential biomarkers for evaluating disease process and provided novel therapeutic targets in patients with thrombocytopenia.

## Data Availability Statement

The original contributions presented in the study are included in the article/**Supplementary Material**. Further inquiries can be directed to the corresponding author.

## Ethics Statement 

The studies involving human participants were reviewed and approved by Fujian Medical University Union Hospital. The patients/participants provided their written informed consent to participate in this study.

## Author Contributions

YWC and LL collected samples, performed the experiments and analyzed data. YZ, NZ, DG and SK provided technical support. QZ analyzed data. ZL coordinated the study. QS and LF made comments to the manuscript. JH supervised the project. YYC initiated and guided the study, interpreted the results, wrote and submitted the manuscript. All authors approved the final version of the manuscript.

## Funding

This study was supported by Joint Funds for the Innovation of Science and Technology in Fujian province (2020Y9056), National Key Clinical Specialty Discipline Construction Program (2021-76) and Fujian Provincial Clinical Research Center for Hematological Malignancies (2020Y2006).

## Conflict of Interest

The authors declare that the research was conducted in the absence of any commercial or financial relationships that could be construed as a potential conflict of interest.

## Publisher’s Note

All claims expressed in this article are solely those of the authors and do not necessarily represent those of their affiliated organizations, or those of the publisher, the editors and the reviewers. Any product that may be evaluated in this article, or claim that may be made by its manufacturer, is not guaranteed or endorsed by the publisher.

## Supplementary Material

Supplementary Table 1 The characteristics and variables in ITP patients and healthy controls for platelet apoptosis assessment.

Supplementary Table 2 The characteristics of representative ITP patients and healthy controls for Western blotting analysis.

Supplementary Table 3 Comparison of desialylation and TFH levels based on sex difference in thrombocytopenia cohort and healthy controls.

Supplementary Figure 1 Patients (ITP, MDS and AA) with high levels of desialylation exhibit impaired megakaryocyte differentiation and maturation in bone marrow. **(A)** Representative Wright-Giemsa-stained bone marrow (BM) sections from patients with different thrombocytopenia and healthy subjects. **(B)** Analysis of BM megakaryocyte counts per field under microscopy. **(C) **Analysis of different stages of megakaryocytes in Wright-Giemsa-stained BM smears from ITP group and healthy controls.

Supplementary Figure 2 Platelet desialylation correlates with therapeutic response in patients with ITP. **(A, B)** Comparison of platelet desialylation levels between ITP patients (platelet count ≥ 150×10^9^/L *vs.* platelet count < 150×10^9^/L) and healthy controls. **(C, D)** Correlation analysis between platelet desialylation and platelet count in entire ITP cohort as determined by Spearman’s test. **(E, F)** ECL and RCA-I levels in complete responders and non-responders. **(G)** Platelet count at each scheduled visit during 12 months follow-up.
