# Supplementary material for: Association of Platelet Desialylation and Circulating Follicular Helper T Cells in Patients With Thrombocytopenia
Source: Front Immunol. 2022 Apr 1;13:810620. doi: 10.3389/fimmu.2022.810620 (PMC9016750; doi:10.3389/fimmu.2022.810620)
Supplement: Supplementary Table 1 — The characteristics and variables in ITP patients and healthy controls for platelet apoptosis assessment. [file Table_1.docx]

**TABLE S1**∣The characteristics and variables in ITP patients and healthy controls for platelet apoptosis assessment

| Characteristics | ITP(n=50) Healthy controls(n=21) P-value |
| --- | --- |

Age, y 33[30, 43] 36 [30, 48] 0.68

Female/Male 35/15 18/3 0.16

PLT(×10^9^/L) 50 [20, 93] 244 [204, 277] < 0.00

ECL(%) 2.2 [0.6, 7.3] 1.0 [0.5, 1.1] 0.01

RCA(%) 2.3 [0.6, 6.7] 0.7 [0.2, 1.0] 0.00

Apoptosis(%) 1.7 [0.9, 2.6] 0.9 [0.6, 1.5] 0.02

CD4+CXCR5+TFHs(%) 6.2 [3.3, 10.1] 2.4 [0.6, 5.2] 0.00

CD4+CXCR5+PD1+TFHs (%) 2.5 [1.0, 5.2] 0.7 [0.4, 1.9] 0.00

CXCL13(pg/ml) 51 [10, 98]^a^  45 [2, 77] ^b^ 0.49

*PLT, platelet; ITP, immune thrombocytopenia; ^a^34 samples only; ^b^11 samples only.*
