# Supplementary material for: Association of Platelet Desialylation and Circulating Follicular Helper T Cells in Patients With Thrombocytopenia
Source: Front Immunol. 2022 Apr 1;13:810620. doi: 10.3389/fimmu.2022.810620 (PMC9016750; doi:10.3389/fimmu.2022.810620)
Supplement: Supplementary Table 2 — The characteristics of representative ITP patients and healthy controls for Western blotting analysis. [file Table_2.docx]

**TABLE S2**∣The characteristics of representative ITP patients and healthy controls for Western blotting analysis

| Characteristics | ITP Healthy controls  #1 #2 #3 #4 #1 #2 |
| --- | --- |

PLT(×10^9^/L) 88 31 11 46 284 228

ECL(%) 0.40 2.95 10.6 36.1 0.96 1.64

RCA(%) 0.67 6.78 6.92 1.75 0.62 1.01

CD4+CXCR5+TFHs(%) 2.55 10.30 14.0 7.12 2.35 0.60

CD4+CXCR5+PD1+TFHs (%) 4.44 5.24 8.04 3.86 3.03 0.69

CXCL13(pg/ml) 28.35 72.81 378.5 93.50 0.67 3.46

PLT: platelet; ITP: immune thrombocytopenia
