# Supplementary material for: Association of Platelet Desialylation and Circulating Follicular Helper T Cells in Patients With Thrombocytopenia
Source: Front Immunol. 2022 Apr 1;13:810620. doi: 10.3389/fimmu.2022.810620 (PMC9016750; doi:10.3389/fimmu.2022.810620)
Supplement: Supplementary Table 3 — Comparison of desialylation and TFH levels based on sex difference in thrombocytopenia cohort and healthy controls. [file Table_3.docx]

**TABLE S3**∣Comparison of desialylation and TFH levels based on sex difference in ITP cohort and healthy controls

|  | Female n Male n P-value |
| --- | --- |

**ITP**

ECL(%) 1.5 [0.4, 6.3] 128 2.9 [0.9, 12] 61 0.05

RCA(%) 3.2 [1.0, 10] 129 6.1 [1.6, 20] 60 0.10

CD4+CXCR5+ TFHs(%) 4.0 [2.0, 8.8] 83 3.6 [1.5, 6.8] 42 0.18

CD4+CXCR5+PD+TFHs(%) 3.1[1.1, 7.8] 80 1.6 [1.0, 4.7] 41 0.07

**Healthy controls**

ECL(%) 0.8 [0.3, 1.0] 74 0.9 [0.7, 1.0] 34 0.40

RCA(%) 0.8 [0.3, 1.0] 75 1.0 [0.5, 1.0] 34 0.32

CD4+CXCR5+ TFHs(%) 1.2 [0.5, 2.5] 51 1.9 [0.7, 3.8] 24 0.33

CD4+CXCR5+PD+TFHs(%) 0.8[0.3, 1.9] 51 1.9 [0.8, 2.0] 23 0.06

*ITP, immune thrombocytopenia.*
