# Supplementary figures and images for: Association of Platelet Desialylation and Circulating Follicular Helper T Cells in Patients With Thrombocytopenia
Source: Front Immunol. 2022 Apr 1;13:810620. doi: 10.3389/fimmu.2022.810620 (PMC9016750; doi:10.3389/fimmu.2022.810620)

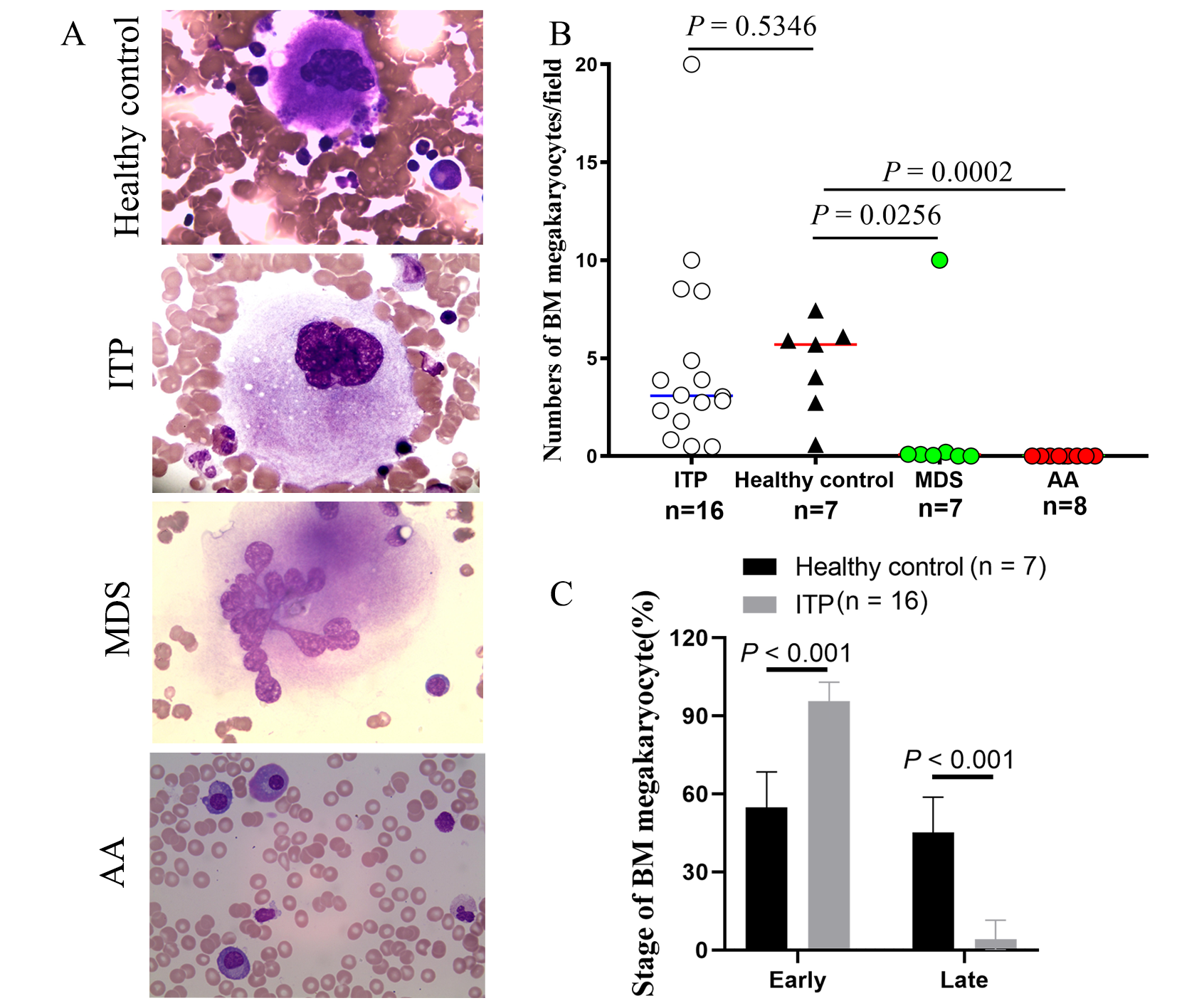

Supplement: Supplementary Figure 1 — Patients (ITP, MDS and AA) with high levels of desialylation exhibit impaired megakaryocyte differentiation and maturation in bone marrow. (A) Representative Wright-Giemsa-stained bone marrow (BM) sections from patients with different thrombocytopenia and healthy subjects. (B) Analysis of BM megakaryocyte counts per field under microscopy. (C) Analysis of different stages of megakaryocytes in Wright-Giemsa-stained BM smears from ITP group and healthy controls. [file Image_1.tif]

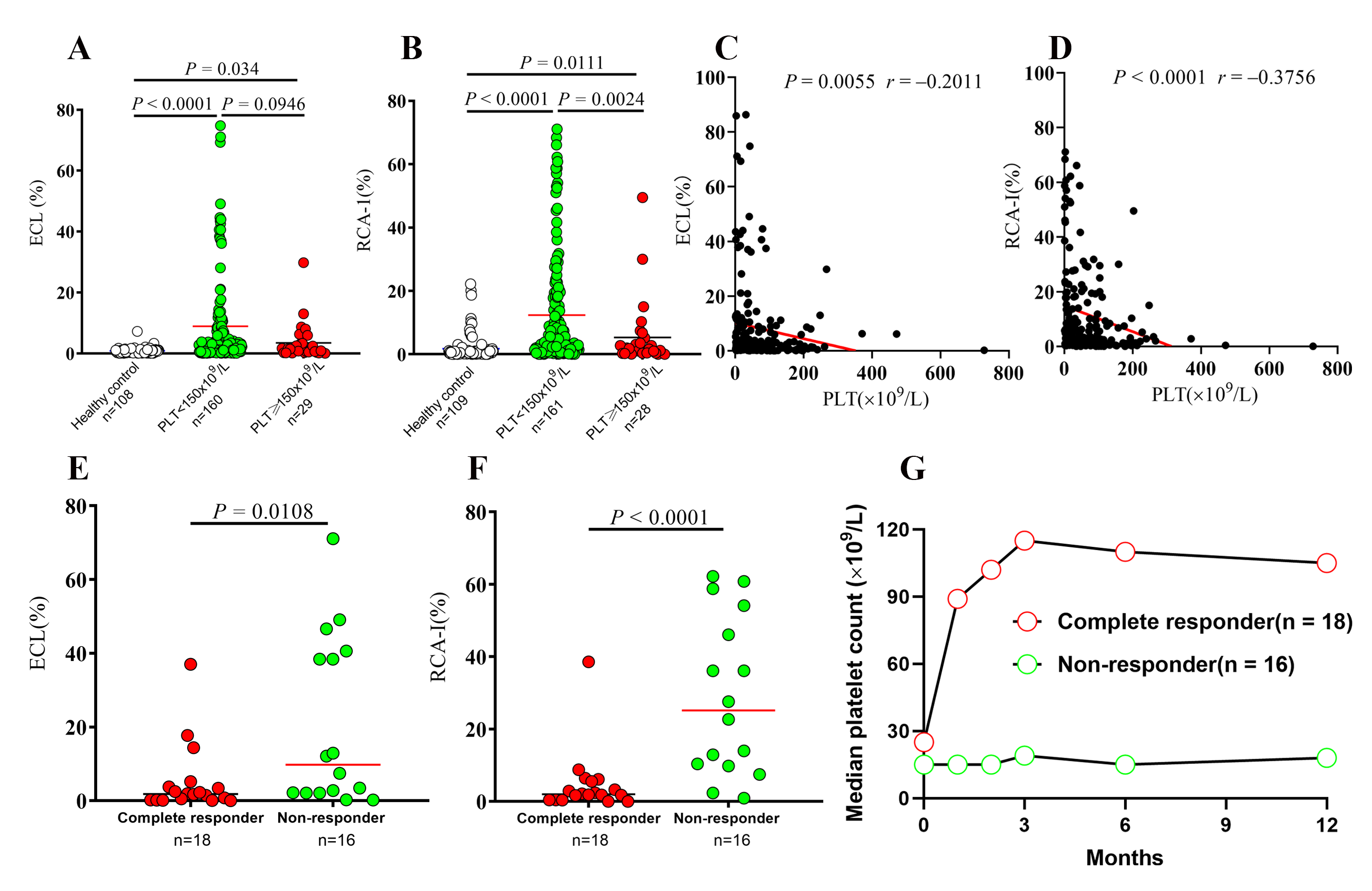

Supplement: Supplementary Figure 2 — Platelet desialylation correlates with therapeutic response in patients with ITP. (A, B) Comparison of platelet desialylation levels between ITP patients (platelet count ≥ 150×109/L vs. platelet count < 150×109/L) and healthy controls. (C, D) Correlation analysis between platelet desialylation and platelet count in entire ITP cohort as determined by Spearman’s test. (E, F) ECL and RCA-I levels in complete responders and non-responders. (G) Platelet count at each scheduled visit during 12 months follow-up. [file Image_2.tif]
